# Negative Online Ratings of Joint Replacement Surgeons: An Analysis of 6,402 Reviews

**DOI:** 10.1016/j.artd.2021.05.005

**Published:** 2021-06-15

**Authors:** Casey Imbergamo, Andrzej Brzezinski, Aneesh Patankar, Matthew Weintraub, Natale Mazzaferro, Stephen Kayiaros

**Affiliations:** a Department of Orthopaedic Surgery, Rutgers Robert Wood Johnson Medical School, New Brunswick, NJ, USA; b Biostatistics and Epidemiology Services Center, Rutgers School of Public Health, Rutgers University, Piscataway, NJ, USA; c Department of Biostatistics and Epidemiology, Rutgers School of Public Health, Rutgers University, Piscataway, NJ, USA

**Keywords:** Online, Ratings, Reviews, Patient, Satisfaction, Arthroplasty

## Abstract

**Background:**

With the expanding accessibility of online health-care information, patients frequently report visiting physician rating websites before choosing a surgeon. As such, it is important to analyze patients’ perception of arthroplasty surgeons as reflected on physician rating websites.

**Methods:**

A total of 6402 online reviews of arthroplasty surgeons were extracted for analysis. Each review rated less than 5 on a 5-point scale was deemed a “negative” review and was subsequently assigned to an appropriate category. Reviews were stratified by practice type, years in practice, gender, and low (<3) vs high (> 3) ratings.

**Results:**

A total of 6402 reviews comprising 315 physicians were included in the analysis. The average rating for all surgeons was 4.35. The average rating for physicians in private practice was 4.3, compared to 4.5 for those in an academic setting. The average rating for physicians in practice for 1-10 years was 4.46, compared to 4.03 for those with >10 years of experience (*P* < .001). The most common factors contributing to negative reviews were bedside manner, wait time, poor outcome, and surgeon proficiency. Surgeon-dependent factors were more commonly associated with lower rated reviews (*P* < .001).

**Conclusions:**

Arthroplasty surgeons typically receive high online ratings, with a mean of 4.35 on a 5-point scale. Physicians in academic practice received higher ratings than those in private practice, and physicians who have been in practice for 1-10 years received higher ratings than those with more than 10 years in practice. The most common factors contributing to negative reviews are surgeon-dependent, including bedside manner, poor outcome, and surgeon proficiency.

## Introduction

Health information is more accessible than ever before, with 59% of all US adults and 75% of adult internet users searching for health information online [1].The accessibility of this information empowers patients to make informed decisions as it broadly relates to their medical care [2]. With the advent and subsequent expansion of public physician rating sites, more patients are relying on reviews and comments to choose a physician for their care, with 28% of consumers searching the internet for information on provider quality [3]. It has been reported that more than 50% of patients who use public physician rating websites considered the information online as important in choosing a physician, and 37% reported that they avoided a physician based on negative reviews [4]. Therefore, it is important for physicians to reflect on these reviews to gauge perceived patient experience. This has the dual purpose of benefiting the patient while also providing invaluable information to improve and grow one’s practice.

The purpose of this study was to evaluate online ratings of joint replacement surgeons using 3 major rating websites, to identify variables of significance that contribute to negative reviews, and to analyze differences in ratings between surgeons by practice setting, years in practice, and gender.

## Material and methods

In June 2020, a Google search was conducted for “physician rating websites.” From the results of this search, 3 sites were identified for use in this study: Healthgrades.com (Denver, CO), Vitals.com (Lyndhurst, NJ), and RateMDs.com (San Jose, CA). Using Google Trends website traffic data, these were identified as the websites with most traffic in 2020 which included patient-generated comments that could be analyzed for the purpose of this study. In addition, these websites were correlated with previous analyses of online reviews of orthopedic surgeons in the existing literature [5,6]. To compile our list of orthopedic surgeons to analyze, we restricted our search to surgeons in the greater New York metropolitan area, including those within a 50-mile radius of New Brunswick, NJ. We then confirmed that all surgeons included were active members of the American Association of Hip and Knee Surgeons (AAHKS), which ensures that they are board-certified and complete at least 50 arthroplasty procedures or osteotomies annually.

A code using Visual Basic for Applications (Microsoft, Redmond, WA), a Microsoft Excel-based programming language, was developed to automate the process of compiling each surgeon’s information from the 3 physician rating websites into Microsoft Excel (Redmond, WA). Data of interest included surgeon name, overall rating, patient reviews, and rating associated with patient review, among other metrics. Three hundred and fifteen surgeons throughout the greater New York metropolitan area were included in the study, all of whom were members of AAHKS, with a total of 6402 ratings. All ratings included were from 2005 to 2020.

Three independent reviewers conducted the analysis of the patient-generated comments. All ratings less than 5 on a five-point scale were marked as “negative” ratings, and the associated comment was subsequently reviewed. Based on the content, the negative review was attributed to one or more of the following categories: ease of scheduling, time spent with patient, staff interaction, wait time, bedside manner, poor outcome, surgeon proficiency, or other. Comments were assigned the category of “other” if they did not definitively fit into one of the aforementioned categories. If there was any uncertainty in the categorization of a comment, a majority decision was reached by the 3 reviewers to assign the comment to an appropriate category.

After completing the review of all 315 surgeons and 6402 reviews, the reviewers subsequently recorded the type of practice for each surgeon as well as their gender. This was performed by completing a Google search for each physician and determining if they had a web page associated with an academic medical center, a medical school, or an orthopedic surgery residency program. If no such web pages existed, the physician was assigned to the private practice category. Surgeons who had a combined private practice with an academic appointment were assigned to the academic practice group. The variables of practice setting, years in practice, and gender were chosen to be evaluated in this study, as these metrics were previously studied in the literature [6]. The null hypothesis was that there would be no significant difference in surgeon rating or composition of negative reviews by practice setting, years in practice, or surgeon gender.

All study measures were first summarized using mean and standard deviation for continuous measures and frequencies with percentages for categorical and ordinal measures. Separate bivariate analyses were performed to compare study measures by physician practice type, website of origin, and a combination of both practice type and website for both individual ratings (n = 6402) and among unique physicians (n = 315). These comparisons were performed using Pearson’s Chi-Square, Fisher’s exact, and independent t-tests. Statistical analyses were performed using R, and all *P* values are two-sided, where <0.05 was considered statistically significant [7].

## Results

A total of 315 physicians were included in the study. One hundred ninety-seven physicians were listed on Vitals, 181 on HealthGrades, and 199 on RateMDs. Of all, 47.1% of surgeons appeared on one website, 28.7% of surgeons appeared on 2 websites, and 24.2% appeared on all 3 websites. There were 309 (98.1%) male and 6 (1.9%) female surgeons. Two hundred twenty-six surgeons (71.7%) were in private practice, and 89 (28.3%) were affiliated with an academic medical center, medical school, or orthopedic surgery residency program. Sixty-seven (21.3%) physicians had been in practice for 1-10 years (average, 2.58), and 248 (78.7%) had been in practice for more than 10 years (average, 27.0) (Table 1).

**Table 1:** Demographics of surgeons included in the study.

Physician gender, years in practice	Academic (n = 89)	Private (n = 226)	Total (n = 315)
Female, n (%)	2 (2.2)	4 (1.8)	6 (1.9)
Male, n (%)	87 (97.8)	222 (98.2)	309 (98.1)
1-10 y, n (%)	26 (29.2)	41 (18.1)	67 (21.3)
>10 y, n (%)	63 (70.8)	185 (81.9)	248 (78.7)

**Figure 1:**
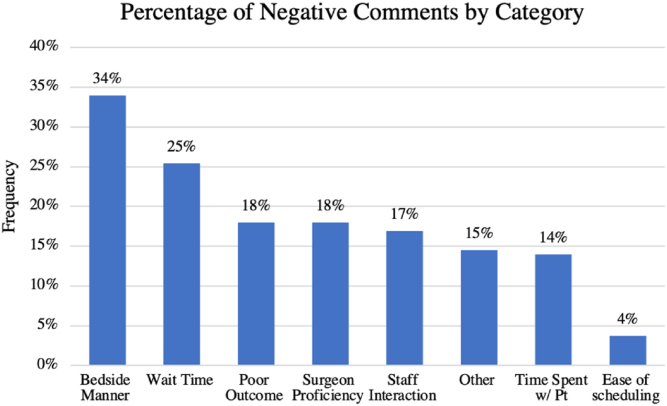
Percentage of negative comments by category (all websites).

A total of 6402 ratings, including 1686 (26.3%) negative comments, were included in the study (Table 2, Table 3). Overall, there was an average of 27.7 ± 24.2 ratings per physician. The average individual rating for physicians across all 3 websites was 4.35 ± 1.4, with surgeons in a private practice rated 4.3 ± 1.4, including 28.6% negative ratings, compared to 4.5 ± 1.3 for those in an academic setting, with 19.2% negative ratings (*P* < .001). The average rating for physicians in practice for less than 10 years was 4.46 ± 0.64 with 12% negative ratings, vs 4.03 ± 0.67 for those in practice for more than 10 years with 28.9% negative ratings (*P* < .001). The sample size of female surgeons included in this study was not adequately powered to complete subgroup analyses. As each negative patient review could encompass more than one categorical complaint (ie, Patient may complain of excessive wait time and poor bedside manner in the same comment.), there were a total of 2386 categorical assignments for 1686 comments (Fig. 1).

**Table 2:** Number of negative ratings by website, practice type, gender, years in practice.

Website	Academic practice ratings (n = 1546)	Private practice ratings (n = 4856)	Total (n = 6402)
HealthGrades, n (% of total ratings)	51 (9.7)	235 (14.3)	286 (13.2)
RateMDs, n (% of total ratings)	98 (32.9)	803 (48.5)	901 (46.1)
Vitals, n (% of total ratings)	148 (20.3)	351 (22.5)	499 (21.8)
Gender			
Female, n (% of total ratings)	5 (17.9)	16 (72.7)	21 (42)
Male, n (% of total ratings)	292 (19.2)	1373 (28.4)	1665 (26.2)
Years in practice			
1-10 y, n (% of total ratings)	44 (10.6)	74 (13)	118 (12)
>10 y, n (% of total ratings)	253 (22.3)	1313 (30.6)	1566 (28.9)

**Table 3:** Number of ratings by website, practice type, gender, years in practice.

Website	Academic practice ratings (n = 1546)	Private practice ratings (n = 4856)	Total (n = 6402)
HealthGrades, n (%)	522 (33.8)	1640 (33.8)	2162 (33.8)
RateMDs, n (%)	297 (19.2)	1656 (34.1)	1953 (30.5)
Vitals, n (%)	727 (47.0)	1560 (32.1)	2287 (35.7)
Gender			
Female, n (%)	28 (1.8)	22 (0.5)	50 (0.8)
Male, n (%)	1518 (98.2)	4834 (99.5)	6352 (99.2)
Years in practice			
1-10 y, n (%)	414 (26.8)	569 (11.7)	983 (15.4)
>10 y, n (%)	1132 (73.2)	4287 (88.3)	5419 (84.6)

Of the 2386 total categorical assignments for negative comments, the most frequent patient complaint was poor bedside manner (34%). In many instances, patients directly referenced “poor bedside manner” in their reviews; however, comments in this category also included statements such as that the physician was “rude”, “dismissive”, “curt”, “demeaning”, or “condescending”. The second most frequently cited reason for a negative review was wait time (25.4%). Many patients documented their frustrations with not being seen at the time which their appointment was scheduled. Patients reported wait times ranging from 30 minutes to 2 hours before being seen by a physician. Poor outcome and surgeon proficiency both comprised 18% of negative reviews. The poor outcome category encompassed things such as postoperative complications or unsatisfactory results after surgery (eg, limited range of motion, continued pain, and so on). Poor surgeon proficiency was noted as a reason for a negative review when patients stated that they perceived the surgeon to be incompetent, lack expected knowledge, or they received what they believed to be an inaccurate diagnosis. Issues with staff interaction were cited in 16.9% of negative reviews. This commonly included instances in which patients had unsatisfactory encounters with staff members over the phone or at the front desk of the physician’s office.

Many patient complaints fell into the other category (14.5%), which included comments that did not specify a reason for their negative review, and only left general remarks such as “Bad doctor” or “Would not come back to this doctor’s office”. There were also comments that cited insurance or billing issues as the main complaint or being seen by a different physician than they originally anticipated. The amount of time spent with the patient was noted in 14% of negative reviews. Patients reported that the physician only spent a few minutes speaking to and examining them or it seemed like they were in a rush to get out of the examination room. Ease of scheduling was only noted in 3.7% of negative reviews. These comments included complaints of limited physician availability or having to wait weeks or months to be able to schedule an appointment.

When dividing negative reviews into surgeon-dependent (time spent with patient, bedside manner, poor outcome, surgeon proficiency) and surgeon-independent (ease of scheduling, staff interaction, wait time) factors, it was found that 41.8% of negative comments cited complaints that were surgeon-dependent only, 26.5% cited complaints that were surgeon-independent only, and 14.3% cited a combination of both for a negative review. The remaining 17.4% of negative reviews had no specific complaint and were categorized accordingly.

When stratifying negative reviews by surgeon-dependence and practice type, it was found that there were significantly more negative reviews citing surgeon-dependent factors in the academic practice group (49.8% vs 39.8%, *P* = .015). Conversely, there were significantly more negative reviews attributed to surgeon-independent factors in the private practice group (27.3% vs 21.9%, *P* = .023).

Physicians in practice for more than 10 years generated more negative reviews pertaining to surgeon-independent factors (26.9% vs 20.0%, *P* = .045), specifically regarding wait time (26.4% vs 12.7%, *P* = .001), than their counterparts with fewer than 10 years in practice. There was no significant difference in surgeon-dependent factors between the 2 groups.

**Figure 2:**
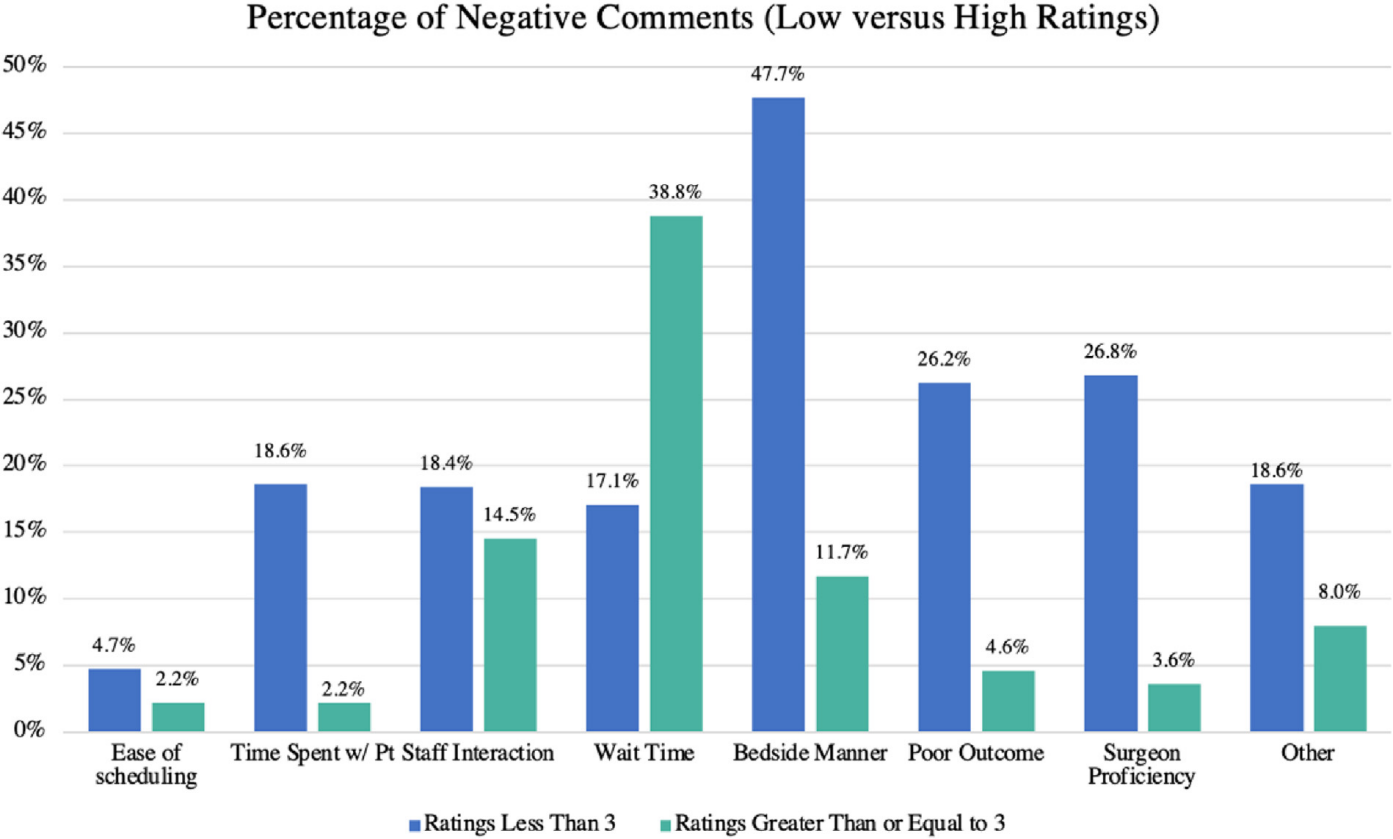
Percentage of negative comments by category (low vs high ratings).

When stratifying negative reviews by high- (> 3) vs low-rated comments (<3), it was found that significantly more low-rated reviews cited surgeon-dependent factors (48.4% vs 13.0%, *P* < .001), while higher rated reviews cited surgeon-independent complaints (51.6% vs 34.1%, *P* < .001). Low-rated reviews also had significantly more comments that cited both surgeon-dependent and surgeon-independent factors (21.1% vs 3.3%, *P* < .001) (Fig. 2).

While this study considered any rating less than 5 on a 5-point scale to be a “negative” review, more than 58% of private physicians and 74.2% of academic physicians received a rating of 4 or greater. The overall distribution of physician ratings between the 2 groups was not statistically significant (*P* = .07) (Table 4). When comparing physicians by years in practice, 76% of physicians with 1-10 years of experience received a rating of 4 or greater, compared to 58.5% in the group with greater than 10 years of experience. In addition, 25.4% of physicians with fewer years in practice received a 5/5 rating, compared to only 4% of those with more than 10 years in practice. The overall distribution of physician ratings between these 2 groups was statistically significant (*P* < .001) (Table 4).

**Table 4:** Physician overall rating by practice setting and years in practice.

Physician overall rating	Academic practice, n (%)	Private practice, n (%)	1-10 Y, n (%)	>10 Y, n (%)
1-1.99	0 (0.0)	3 (1.3)	0 (0.0)	3 (1.2)
2-2.99	2 (2.2)	15 (6.6)	3 (4.5)	14 (5.6)
3-3.99	21 (23.6)	75 (33.2)	10 (14.9)	86 (34.7)
4-4.99	59 (66.3)	113 (50.0)	37 (55.2)	135 (54.4)
5	7 (7.9)	20 (8.8)	17 (25.4)	10 (4.0)

## Discussion

This study evaluated 6402 reviews of joint replacement surgeons using 3 major physician rating websites: Vitals, HealthGrades, and RateMDs. Variables of significance which contributed to negative reviews were identified, and results were stratified by practice setting and years of experience. Overall, reviews for joint replacement surgeons were positive, with an average rating of 4.35 ± 1.4 on a 5-point scale. Patients were generally satisfied with their experiences, and of the 6402 reviews included in the study, only 1686 (26.3%) were rated less than 5. While there was a statistically significant difference in average individual ratings for surgeons in academic (4.5) vs private practice (4.3), the clinical relevance of this is likely negligible as both groups achieved high ratings. The reputation of academic centers being highly specialized centers of excellence may contribute to improved patient perceptions of the surgeons affiliated with them.

This study also found a significant difference in the average individual rating for surgeons in practice for 1-10 years (4.46) vs those in practice for more than 10 years (4.03). In addition, significantly more surgeons with fewer years in practice received a rating of 5 on a 5-point scale (25.4%), compared to only 4% of those with greater years in practice. This difference may potentially reflect a paradigm shift in the training of orthopedic surgeons, with a holistic approach to patient care being emphasized in more recent years, leading to improved patient satisfaction. In addition, many orthopedic practices use marketing tools to solicit positive reviews for younger surgeons from satisfied patients in an effort to help build their practices and earn them a good reputation. Another hypothesis to consider is that younger surgeons are more likely to be active on social media platforms, which might potentially make them more recognizable, leading to improved patient perception. Nwachukwu et al. looked at the correlation of online ratings of sport medicine surgeons and their activities on social media platforms including presence on Facebook, Twitter, and other personal websites, but the authors did not find any statistically significant correlations [5]. A recent study by Runge et al. examining online ratings of AAHKS members also found that fewer years in practice (1-10 years) correlated to higher average ratings, coinciding with the present findings in this study [6]. The authors hypothesized that younger surgeons may potentially spend more time with the patient to better recognize and address the patients’ expectations and more often analyze their online ratings to look at areas of improvement in interactions with patients.

Yu et al. analyzed ratings and written reviews for orthopedists in a single metropolitan area and found that higher scoring reviews were associated with successfully addressing and treating the patient’s original complaint, while factors that drove low ratings included unresolved symptoms, scheduling difficulties, lack of communication regarding care, and lack of compassion [8].

Previous studies have also investigated factors contributing to positive and negative reviews of orthopedic surgeons. When investigating orthopedic surgeons in the St. Louis metropolitan area, Bakhsh and Mesfin found that bedside manner and surgeon proficiency and knowledge most significantly contributed to the overall physician rating [9]. These findings correlated with the results from our study, which also found bedside manner and surgeon proficiency to be among the most common reasons for negative reviews. A study by Kalagara et al. investigated spine surgeons in North America and found that surgeon trustworthiness was the greatest predictor of average rating, while negative comments regarding surgeon competence correlated with lower ratings [10]. Similarly, the results of this study revealed that surgeon-dependent factors were the most cited reasons for negative reviews. The factors contributing to negative reviews of orthopedic surgeons appear to be consistent as demonstrated by multiple studies [6,[11], [12], [13]].

With a focus on value-based care, the Centers for Medicaid and Medicare Services rely on patient-reported outcomes as outlined in the Consensus Core Set of Orthopedic Measures, a collaborative aimed at collecting value-based measurements, to aid in determining reimbursement rates [14]. These physician rating sites certainly are not complete when compared to the comprehensive questions asked in the Consensus Core; however, the information provided by these physician rating sites can still provide valuable insight into patient satisfaction surveys used by Centers for Medicaid and Medicare Services for reimbursement purposes [15]. The categories included in this study were reflective of the patient experience domain items from the Consensus Core Set of Orthopedic Measures, as well as variables previously established in the analysis of online reviews of orthopedic surgeons [9].

Of all negative reviews in this study, the most common complaints pertained to bedside manner. Patients frequently noted that they felt the surgeon was dismissive or condescending. To minimize such complaints, the patient-physician relationship must be addressed, a responsibility that falls directly on surgeons. Notably, it was found that negative comments regarding bedside manner were more common for surgeons in an academic practice setting.

The second most cited category was wait time. Patients stated their frustration with long wait times to see their physician, on occasion reaching a few hours. These complaints may potentially be addressed by evaluating the efficiency of office workflow or adjusting scheduling to ensure that patients are seen within a reasonable timeframe. In this study, it was found that negative comments regarding excessive wait times were more common for surgeons in private practice and those who have been in practice for >10 years; however, it has not been elucidated whether an objective difference in wait times exists among these groups.

The next most common complaints were poor outcome and surgeon proficiency. The unfortunate reality of orthopedics and joint replacement is that poor outcomes, while rare, do occur. Overall complication rates after total joint arthroplasty have been found to be 8% in outpatient settings and 16% in inpatient settings [16]. To address this, it has been found that managing patient expectations regarding outcomes after surgery may potentially improve patient satisfaction after arthroplasty [17]. Regarding surgeon proficiency, it can be considered that spending extra time to explain the diagnosis or treatment of a patient’s condition may alleviate their feelings of dissatisfaction. A recent study by Shen et al. found that the most common questions searched on the internet regarding total joint arthroplasty pertained to arthritis management, rehabilitation, and patients’ ability to perform specific tasks [18]. Addressing these topics with patients may potentially improve satisfaction and alleviate surgeon-dependent complaints pertaining to bedside manner or surgeon proficiency.

In an effort to improve online ratings, some physicians report soliciting favorable reviews from patients. A study by Samora et al. surveyed members of the American Society for Surgery of the Hand and found that 33% of surgeons reported requesting satisfied patients to submit reviews [19]. In addition, this study found that only 3% of respondents reported paying online reputation management companies to improve their online ratings [19]. While ethically questionable, such companies exist that serve the purpose of improving physician online ratings in exchange for a fee. However, it was beyond the scope of the present study to determine whether surgeons solicited positive reviews from satisfied patients or payed third-party companies to manage their ratings.

We found that surgeon-dependent factors were cited more often in lower rated reviews (<3), implying that these factors may have the greatest impact on patient dissatisfaction. Alternatively, among higher rated reviews (>3), patients cited surgeon-independent factors, including wait time, as the main reason for their negative review. While patients may view excessive wait times as a negative aspect of their care, it is less likely to cause substantial dissatisfaction.

To our knowledge, this is the first study to specifically analyze ratings and comments for members of the American Academy of Hip and Knee Surgeons across the greater New York metropolitan area. Both ratings and written reviews were placed into discrete categories to identify factors that most negatively affect patient satisfaction. This information may help physicians target potential areas for improvement in their own practices, which can in turn translate into a better patient experience.

This study was not without limitations. Data were collected from 3 of the most content-dense physician rating websites with heavy traffic; however, there are numerous additional sites which could not feasibly be incorporated into the study. The sample size for female physicians in this study was small, with only 6 physicians and 50 total reviews. It was not possible to complete subgroup analyses by gender or draw significant conclusions from such a small sample size. While the field of orthopedic surgery is predominantly male, a larger online search encompassing a greater geographic area would be required to evaluate any potential differences in online patient feedback between male and female surgeons [20]. Furthermore, patient-generated ratings and comments of physicians on public websites are inherently subjective in nature, and there is no practical way to determine the validity of any individual rating. In addition, it was not feasible to determine whether specific negative reviews pertained to arthroplasty care or generic orthopedic care provided by the surgeon. Many arthroplasty surgeons take general orthopedic call, and patients seen in this setting may leave more negative reviews than those in an arthroplasty care setting, which allows for a stronger surgeon-patient relationship to be established. However, this variable was not able to be specifically assessed in the present study but merits further investigation in future studies.

## Conclusions

Arthroplasty surgeons typically receive high online ratings, with a mean of 4.35 on a 5-point scale. Surgeons in an academic setting and those in practice less than 10 years receive higher ratings. The most common factors contributing to negative reviews are surgeon-dependent, including bedside manner, poor outcome, and surgeon proficiency. While wait time was also cited as a common complaint, it was not found to be associated with lower-rated reviews.

## Conflicts of interest

The authors declare that they have no known competing financial interests or personal relationships that could have appeared to influence the work reported in this article.

## Appendix A Supplementary data

Conflict of Interest Statement for Kayiaros

Conflict of Interest Statement for Brzezinski

Conflict of Interest Statement for Patankar

Conflict of Interest Statement for Imbergamo

Conflict of Interest Statement for Weintraub

Conflict of Interest Statement for Mazzaferro
